# Magnetization Switching in the GdFeCo Films with In-Plane Anisotropy via Femtosecond Laser Pulses

**DOI:** 10.3390/molecules26216406

**Published:** 2021-10-23

**Authors:** Daria O. Ignatyeva, Pavel O. Kapralov, Kiran Horabail Prabhakara, Hiroki Yoshikawa, Arata Tsukamoto, Vladimir I. Belotelov

**Affiliations:** 1 Physics and Technology Institute, V.I. Vernadsky Crimean Federal University, 295007 Simferopol, Russia; belotelov@physics.msu.ru; 2 Russian Quantum Center, 121205 Moscow, Russia; kapralov_pavel@mail.ru; 3 Photonic and Quantum Technologies School, Lomonosov Moscow State University, 119991 Moscow, Russia; 4 Institute for Molecules and Materials, Faculty of Science, Radboud University, 9102 Nijmegen, The Netherlands; kiranhorabail@gmail.com; 5 Department of Electronic Engineering, College of Science and Technology, Nihon University, Tokyo 101-8308, Japan; yoshikawa.hiroki@nihon-u.ac.jp (H.Y.); tsukamoto.arata@nihon-u.ac.jp (A.T.)

**Keywords:** optomagnetism, magnetization switching, in-plane magnetization, ferrimagnets

## Abstract

Ferrimagnetic rare-earth substituted metal alloys GdFeCo were shown to exhibit the phenomenon of all-optical magnetization switching via femtosecond laser pulses. All-optical magnetization switching has been comprehensively investigated in out-of-plane magnetized GdFeCo films; however, the films with the in-plane magnetic anisotropy have not yet been studied in detail. We report experimental observations of the magnetization switching of in-plane magnetized GdFeCo films by means of the femtosecond laser pulses in the presence of a small magnetic field of about 40 µT. The switching effect has a threshold both in the applied magnetic field and in the light intensity.

## 1. Introduction

Growing demand in the fast and compact information storage devices inspires studies of the magnetization switching performed via all-optical or optically assisted means by the laser pulses. Heat-assisted memory recording (HAMR) is one of the examples of such possibilities and is currently used in modern HDDs. Magnetic materials with rather high coercive fields are used to ensure bit stability. At the same time, optical heating of the desired nanoscale area allows for the local reduction of the coercive field and targeted magnetization switching of this area. An ability to focus light at the nanoscale is a key feature for the reduction of the bit size in this case.

At the same time, recent studies show that the ferrimagnetic alloys [1] of GdFeCo exhibit the all-optical magnetization switching and have potential for data storage applications. This effect reveals itself as the deterministic switching of the magnetization to the opposite one at the picosecond timescale [2] in the area of action of a single femtosecond pulse of an arbitrary polarization [3,4,5]. The origin of all-optical switching is the ultrafast dynamics of the exchange-coupled sublattices [6,7] launched by the thermal action of the laser, thus making it a threshold effect [8]. For fluence values lower than the threshold, an ultrafast demagnetization occurs and the initial magnetization state is restored after it [9]. All-optical switching is observed for certain GdFeCo compositions, while in the other cases, thermomagnetic switching occurs [10,11]. Thermomagnetic switching results from the reduction of the coercivity under local laser-induced heating of the magnetic film [12,13,14,15]. In this case, the magnetic field weaker than the coercive one locally changes the magnetization of the illuminated area, while the magnetization of the whole film remains unperturbed.

Therefore, being completely different in nature, both all-optical and thermomagnetic switching mechanisms rely on the optical energy heating the spin system. In contrast to the coherent optomagnetic effects, such as generation of the effective magnetic fields via circularly polarized [16,17,18] or linearly polarized [19,20,21,22] laser pulses, the thermal mechanism makes the magnetization dynamics generally insensitive to the light polarization, wavelength, and wave vector of light unless they change the energy that the laser pulse delivers to the film. This opens a broad range of opportunities for the control of the energy delivered by the laser pulse via the light polarization due to the magnetic circular dichroism [8,23] or interference in the multilayered structures [24,25].

An important problem for optical or optically assisted magnetization switching is the diffraction limit. This prevents light from tightly focusing into the significantly subwavelength area of ~10 nm and thus decreases by ~1000 times the recording density in real storage applications. It has been demonstrated that utilization of the ferrimagnetic nanoantennas allows the remagnetized area to be reduced in size down to 50 nm [26,27]. Multilayered plasmonic structure, in its turn, makes it possible to address the desired plasmonic GdFeCo layer without an impact on the other ones [28]. Excitation of the localized or surface plasmons also enhances the efficiency of the light–matter interaction and lowers the magnetization switching threshold, as was theoretically suggested in [22,29].

Recent studies have shown that magneto-plasmonic structures significantly enhance the efficiency of light interaction with in-plane magnetized films and structures and result in the enhancement of the transverse and longitudinal Kerr magneto-optical effects [30,31,32,33]. This is an important point for the magneto-optical in-plane magnetized bit reading since the magneto-optical effects produced by the smooth films with the in-plane magnetization are extremely small. Appendix A shows how the idea of the plasmonic-boosted transverse magneto-optical Kerr effect could be applied to the studied sample.

It is important to note that most of the studies of all-optical and thermomagnetic switching in GdFeCo and other ferrimagnetic materials focus on films with out-of-plane anisotropy. At the same time, an ability to optically switch the magnetization of GdFeCo alloy with a certain composition and in-plane magnetization was shown recently [3] but was not studied in detail.

In the present work, we study the GdFeCo film with in-plane magnetic anisotropy and experimentally demonstrate optically assisted magnetization switching in the presence of the low 40 µT magnetic field.

## 2. Results and Discussion

### 2.1. Irreciprocal Magnetization Switching of GdFeCo Films via Femtosecond Laser Pulses

**Figure 1:**
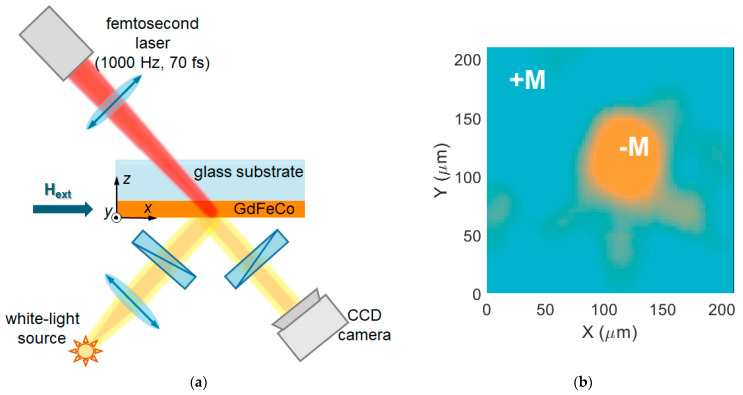
Irreciprocal magnetization switching of the GdFeCo film with in-plane magnetization. (**a**) Scheme of the experimental setup. (**b**) Magnetization state of the GdFeCo film (false-color image) after the exposure to a femtosecond laser pulse of similar parameters: fs-pulse induced switching for +**M** initial state and had no effect for –**M** state.

The experimental scheme is presented in Figure 1a (see the Methods section for details). It comprises the femtosecond laser used for the magnetization switching, a GdFeCo sample with the in-plane magnetic anisotropy, and the white-light set-up for magnetization detection via the longitudinal magneto-optical Kerr effect (LMOKE) measurement. The sample consists of a ${\mathrm{Gd}}_{38}{\mathrm{Fe}}_{54.3}{\mathrm{Co}}_{7.7}$ film of 10 nm thickness deposited on a SiO_2_ glass substrate covered with a 10 nm thick Si_3_N_4_ layer. The GdFeCo layer is preserved from oxidation by another 10 nm thick Si_3_N_4_ layer covering it from above. The femtosecond laser pulse with a fluence of 14 mJ/cm^2^ and a duration of 70 fs is focused on the GdFeCo film in a spot approximately 85 µm in diameter. The sample is magnetized uniformly in the *x*-direction using an electromagnet with a magnetic field of 20 mT (Figure 1a). During the femtosecond pulse action, the magnetic field of the electromagnet was lifted, although the external magnetic field of Earth (shown as $H_{\mathrm{ext}}$ in Figure 1a), oriented in the *x*-direction and having a measured value of 40 µT, had an impact on the sample. The magnetization state of the GdFeCo film was controlled via LMOKE measurement using post-processing of CCD camera images (see the Methods section for the details).

We carried out experiments for the two opposite initial orientations of magnetization and observed that for +**M** magnetization, the femtosecond pulse caused the magnetization switching of the illuminated area to the opposite direction, while the –**M** magnetized state did not switch under the action of the femtosecond pulse with similar characteristics. This means that the mechanism of all-optical switching that is observed for the out-of-plane magnetized GdFeCo films, which was observed for some types of the in-plane GdFeCo films, is not involved in this case. On the contrary, the revealed mechanism of magnetization switching of the studied in-plane magnetized GdFeCo film is heat-assisted magnetization switching in low external magnetic fields of ~50 μT.

To understand the nature of the observed effect, a more detailed study was performed to reveal how the process of magnetization switching depends on the sample orientation, femtosecond pulse characteristics, and external magnetic field.

### 2.2. Impact of the Femtosecond Pump Parameters on the Irreciprocal Magnetization Switching Process

To ensure the thermal nature of the observed magnetization switching and its irreciprocity, we varied the parameters of the femtosecond pulse and studied the process of magnetization switching. The size of the remagnetized area was measured to qualitatively characterize the effect.

**Figure 2:**
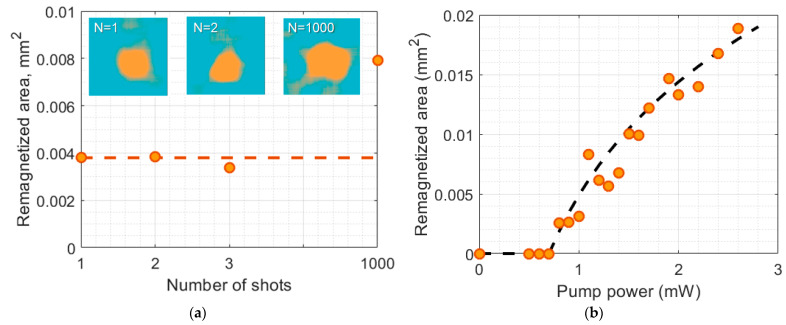
Thermal mechanism of magnetization switching in the GdFeCo film with in-plane magnetic anisotropy. (**a**) The dependence of the remagnetized area on the number of femtosecond laser shots. The insets (false-color) show the magnetization state after the action of the train of femtosecond pulses containing *N* = 1, 2 and 1000 shots. (**b**) The dependence of the remagnetized area on the power of the femtosecond laser pulse trains.

The important feature of the observed magnetization switching is that it is observed after a single-shot exposure. At the same time, the remagnetized spot is not erased and keeps its size via further exposure of the GdFeCo film to the second, third, etc. pulse (see Figure 2a). This is in contrast to the out-of-plane GdFeCo films exhibiting all-optical magnetization reversal after each laser shot due to the peculiar sublattice dynamics and interaction. The total remagnetized area shown in Figure 2a slightly grows after each shot due to the spatial fluctuations of the pulse position. For further experiments, *N* = 1000 shots were used to minimize the impact of the fluctuations of the laser intensity and spatial distribution on the remagnetized area size.

The observed magnetization switching effect was independent of the polarization and the angle of incidence of the femtosecond pulse (see Appendix B for the details). The independence of the phenomenon on the relative directions of the **k**-vector of light, its polarization **E,** and film magnetization **M** prove the absence of coherent optomagnetic effects, e.g., the inverse Cotton–Mouton effect and photoinduced magnetic anisotropy. On the contrary, the magnetization switching that takes place in a ferrimagnetic film heated by the laser depends on the direction and magnitude of the external magnetic field and pulse fluence due to the thermal action of the femtosecond pulse and variation of the coercive field. The thermally assisted magnetization switching is a threshold mechanism since the coercive field decreases with the temperature increase $H_{c}\left(T\right)$. As the GdFeCo film temperature increases locally due to the femtosecond pulse absorption, this means that there is a threshold level of the absorbed energy providing the magnetization switching.

We assume the femtosecond pulse to have a Gaussian spatial distribution of fluence $F\left(\rho \right)=\exp\left(-\frac{4\ln2\;\rho^{2}}{d^{2}}\right)$, where ρ is a polar radial distance in the *x-y* plane and *d* is the beam diameter determined as the boundary of the ½ of the maximum fluence level. The remagnetized area $S_{\mathrm{rm}}$ determined by the condition $F\left(\rho \right)\geq F_{t}$ can be estimated as follow Equation (1):

(1) $$S_{\mathrm{rm}}=\frac{\pi d^{2}}{4\ln2}\ln\frac{F_{0}}{F_{t}}=\frac{\pi d^{2}}{4\ln2}\ln\frac{W_{\mathrm{inc}}}{W_{t}},$$

where $W_{\mathrm{inc}}$ is the incident laser pulse power and $W_{t}$ is the threshold power determined as $W_{j}=\pi d^{2}/\left(4\ln2\right)F_{j}$. Figure 2b shows the experimentally obtained dependence $S_{\mathrm{rm}}\left(W_{\mathrm{inc}}\right)$ that is in good correspondence with the fit by Equation (1). The threshold of magnetization switching is observed at the laser power of 0.7 mW that corresponds to the fluence of $F_{0}=$ 5.1 mJ/cm^2^.

With the increase in the pulse energy, the overpassing of the threshold fluence is observed for a larger area of the beam. Thus, further increases in fluence results in the increase in the area remagnetized after the pulse action for +**M** initial magnetization state and still provides zero impact on the –**M** magnetized state that was also controlled in the experiment. When the laser fluence reaches $F_{\mathrm{inc}}=22\;\mathrm{mJ}/{\mathrm{cm}}^{2}$, the damage to the film occurred for both initial magnetization states.

### 2.3. The Impact of the In-plane GdFeCo Film Anisotropy

**Figure 3:**
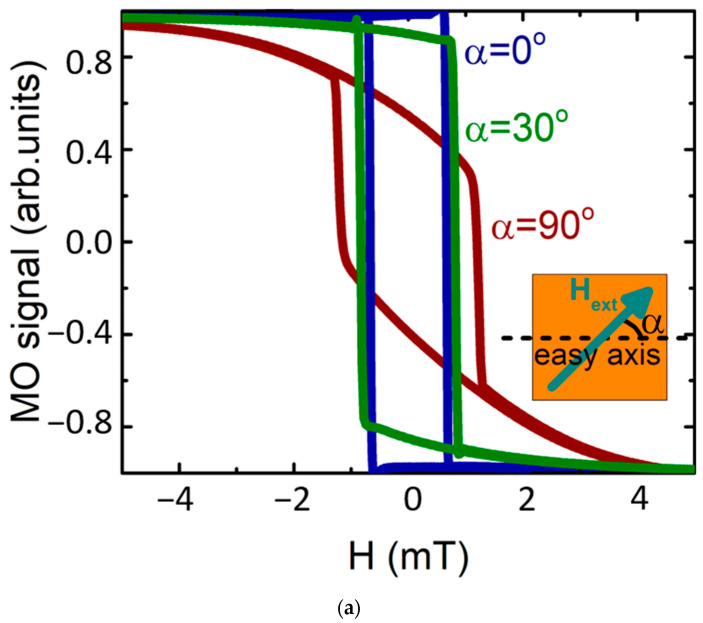

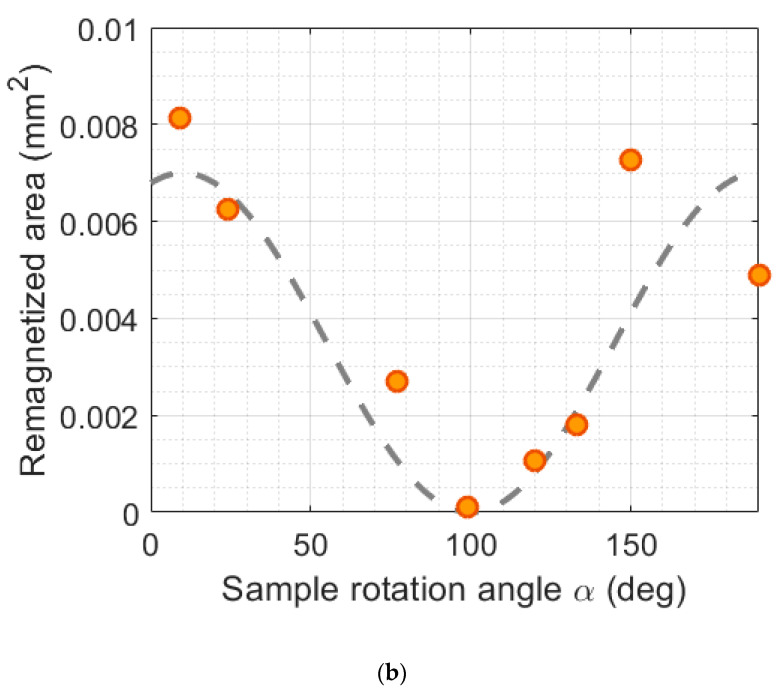
In-plane anisotropy of GdFeCo magnetization switching. (**a**) Hysteresis loops for different angles α describing relative orientations of applied external magnetic field **H** and easy axis of GdFeCo film. The inset shows the studied configuration. (**b**) Dependence of the femtosecond pulse-assisted remagnetized area on the orientation of the initial magnetization (orange dots). The gray line is a guide to the eye and shows cos^2^ dependence.

The anisotropy of the film plays a crucial role in the heat-assisted magnetization switching process. The studied GdFeCo film has the in-plane type of magnetic anisotropy. The hysteresis loops corresponding to different angles α between the applied external magnetic field (x-axis) and anisotropy axis of the GdFeCo film are shown in Figure 3a. The MO signal was measured due to the transverse magneto-optical Kerr effect (TMOKE) as the difference in the reflectance for p- and s-polarized light at the oblique incidence in the transverse configuration (plane of light incidence was set perpendicular to the magnetic field). In such a configuration, the MO signal was proportional to the $M_{x}$ component, while the measured saturation magnetization was $M_{s}=$ 200 kA/m. The magnetic response of the film is highly anisotropic in the film plane. There are two perpendicular in-plane directions characterized by the three-times different saturation magnetic fields 0.4 mT (α=0°) and 1.2 mT (α=90°), correspondingly, and 3-times different saturation magnetization, causing the difference in the MO signal for these orientations. For all directions of the in-plane magnetic field, the remanent magnetization (at *H* = 0) almost equals the saturated one, which proves the in-plane character of the magnetic anisotropy.

The in-plane anisotropy of the magnetic film causes a significantly anisotropic response to the irradiation of the film by the femtosecond laser pulse. If the film is initially magnetized along the easy axis ($\alpha =0^{\circ }$), then the magnetization switching via the femtosecond laser pulse is most efficient: the remagnetized area is the largest (Figure 3b). At the same time, for the orthogonal initial state (along $\alpha =90^{\circ }$), the GdFeCo film is not switched at all. For the optically-assisted switching, the saturating magnetic field is switched off. The film is exposed only to the weak magnetic field of Earth directed along the x-axis. The angle α between the Earth magnetic field and the easy axis is changed by the film rotation, also at an angle α with respect to the easy axis.

### 2.4. The Impact of the External Magnetic Field

**Figure 4:**
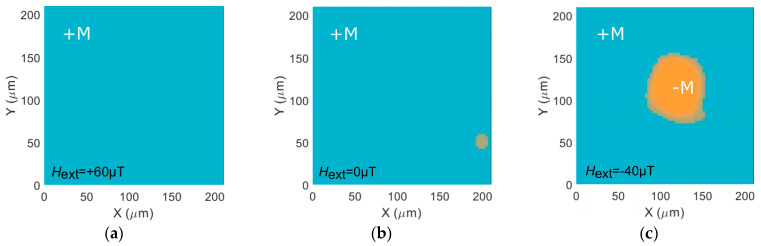

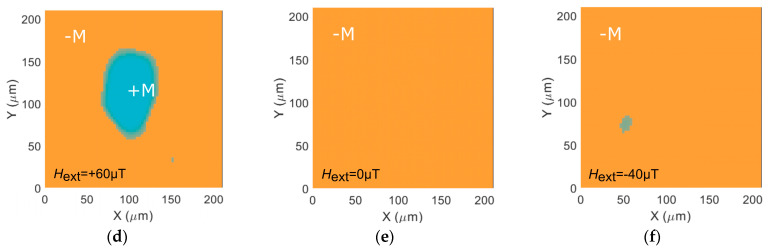
Optical switching of the GdFeCo film under the application of an external magnetic field in the direction of the Earth’s magnetic field and along the easy axis of magnetic anisotropy ($\alpha =0^{\circ }$). Images of the GdFeCo magnetization state after the femtosecond pulse action with (**a**–**c**) +**M** and (**d**–**f**) –**M** initial magnetization under the application of the resulting magnetic field (**a**,**d**) $H_{\mathrm{ext}}=+60\;\mu T$, (**b**,**e**) $H_{\mathrm{ext}}=0\;\mu T$, (**c**,**f**) $H_{\mathrm{ext}}=-40\;\mu T$.

We have also studied the optically induced magnetization switching with the applied small external magnetic field. In the experiments described above, only the magnetic field of the Earth, with a magnitude of 40 µT, influenced the sample. Let us apply an additional external magnetic field of different values along the x-axis to compensate the Earth’s magnetic field and to change the orientation of the total magnetic field to the opposite one. At this, the easy axis of the sample is also oriented along the x-axis ($\alpha =0^{\circ }$). Depending on the orientation of the total external magnetic field applied to the structure, it is possible to switch either +M or –M state (Figure 4), and the remagnetized area size increases for larger magnetic fields: $S_{rm}=0.0062\;{\mathrm{mm}}^{2}$ for $H_{ext}=+60\;\mu T$ and $S_{rm}=0.0049\;{\mathrm{mm}}^{2}$ for $H_{ext}=-40\;\mu T$.

Figure 4 shows that optically induced switching, in this case, takes place only when some external magnetic field is applied. Thus, for H = 0, magnetization is kept (Figure 4b,e). However, the application of a minor external magnetic field makes the difference. An external magnetic field with the magnitude 10 times smaller than the coercive field at the room temperature allows optical switching, and the magnetization is reversed in the direction along the external magnetic field: from –**M** to +**M** for the field along the x-axis (Figure 4d) and from +**M** to –**M** for the opposite external magnetic field (Figure 4c).

## 3. Materials and Methods

### 3.1. Fabrication of the In-Plane GdFeCo Films

Thin 10 nm thick ferrimagnetic amorphous GdFeCo film was deposited by magnetron co-sputtering of Gd, Fe, and Co elements with the direct current (DC) on an atomically flat SiO_2_ substrate. The concentration of alloy compounds was tuned by controlling the relative deposition rates. The ${\mathrm{Gd}}_{38}{\mathrm{Fe}}_{54.3}{\mathrm{Co}}_{7.7}$ composition was chosen to obtain in-plane magnetic anisotropy. Such GdFeCo composition is expected to have a high-temperature compensation point $T_{M}∼450K$ and a Curie temperature of $T_{C}∼600K$ according to the published data [34,35].

### 3.2. Experimental Setup for Magnetization Switching

We used a femtosecond laser (Light Conversion “Pharos”) that emitted single femtosecond pulses of the 800 nm wavelength that were focused on the sample surface by a lens in a spot of 110 um diameter. For the visualization of the film magnetization state, a magneto-optical microscope was used, which consists of a halogen lamp that illuminates the sample surface with a light collimated by a lens and polarized after passing through the polarizer. After reflectance from the sample surface, the light passed through the analyzer oriented at 45 degrees with respect to the polarizer axis to provide linear modulation proportional to the magneto-optical rotation angle. Then, the light was focused using a microscope objective and after passing through the color filter that reduced light intensity, and its spatial distribution was registered using the CCD camera with a 16-bit dynamic range.

### 3.3. Processing of the CCD Images of the Magnetization State

The in-plane film magnetization could be detected via longitudinal (LMOKE) or transversal (TMOKE) magneto-optical Kerr effects. Both LMOKE and TMOKE are significantly lower than the polar Kerr effect usually used for similar studies of out-of-plane magnetization states, and the noise level in our CCD images was high. We used a Gaussian filter with σ=15 μm for the images to remove the high-frequency camera noise. For calculation of remagnetized area, we also filtered the low-amplitude fluctuations of the resulting background.

## 4. Conclusions

Summarizing the results, we have shown that the 10 nm thick ${\mathrm{Gd}}_{38}{\mathrm{Fe}}_{54.3}{\mathrm{Co}}_{7.7}$ film with the in-plane magnetic anisotropy exhibits femtosecond-laser-pulse-induced thermally assisted magnetization switching governed by minor magnetic fields of ~40 µT. The control magnetic field is 10 times smaller than the coercive magnetic field. The demonstrated magnetization switching is a kind of heat-assisted magnetic recording method, though governed with much smaller magnetic fields. The film has the in-plane magnetic anisotropy that opens up additional opportunities for manipulation of the magnetization state by the external magnetic field assisted by a laser pulse.

## Figures and Tables

**Figure A1:**
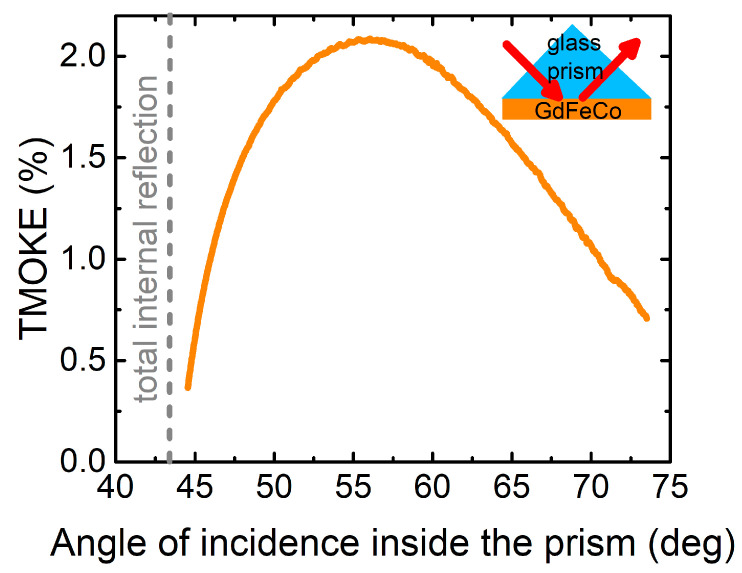
Experimental measurement of the transverse magneto-optical Kerr effect (TMOKE) enhancement near the SPP excitation angle (55 deg) for 800 nm wavelength. The inset shows the measurement configuration.

**Figure A2:**
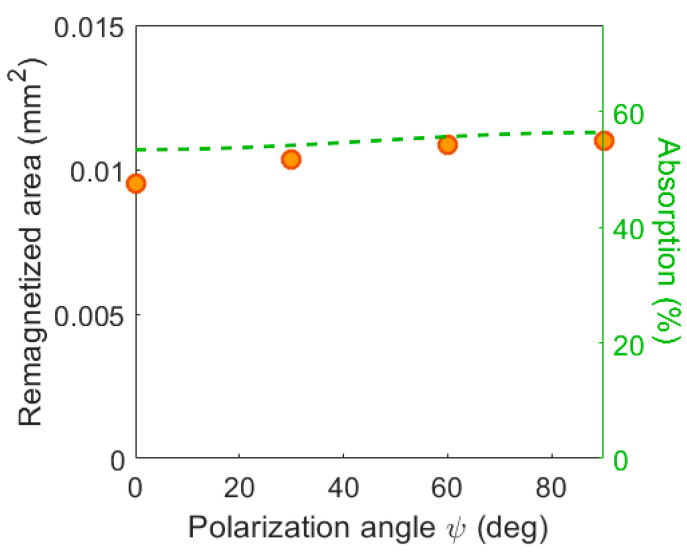
Magnetization switching via femtosecond pulses of different polarization. Experimental measurements of the remagnetized area (left axis) and simulated light absorption (right axis) dependence on the polarization of the femtosecond pulse ψ.

## Data Availability

The data presented in this study are available on request from the corresponding author.

## Sample Availability

Samples of the compounds are available from the authors.

## Appendix A

The scope of our research was to study the optically assisted magnetization switching, which can be possibly used for information writing applications. However, an important point is how one can read written information. Here, we deal with the in-pane magnetized films where both the transverse and longitudinal Kerr effects are extremely small. In order to overcome this problem, we use magneto-optical signal accumulation and averaging over multiple CCD images, which is rather slow for real applications in data reading. Another way is to boost the magneto-optical signal using surface plasmons. Figure A1 shows the TMOKE enhancement near the SPP excitation angle of ~$55^{\circ }$ in the Kretschmann scheme (see the inset). TMOKE was measured for 800 nm wavelength as $\mathrm{TMOKE}=\frac{R\left(+H\right)\;–\;R\left(\;–\;H\right)}{\frac{1}{2}\left(R\left(+H\right)+R\left(\;–\;H\right)\right)}\times 100\%$ with the external magnetic field *H* = 10 mT applied perpendicular to the p-polarized light plane of incidence. TMOKE reaches 2.2% for SPP-supporting GdFeCo+prism structure while for a bare GdFeCo film is 0.15% for the same wavelength and $\theta =30^{\circ }$ angle of incidence.

## Appendix B

To prove that the phenomenon of irreciprocal magnetization switching has thermal nature and is independent of the light pulse parameters except for the fluence, we performed the set of experiments for different angles of incidence$—\theta =0^{\circ },\;15^{\circ },30^{\circ }$—which showed that the observed switching has a constant laser power threshold of $W_{t}=0.7\pm 0.05\;\mathrm{mW}$, and the irreciprocal nature was observed both for the normal and oblique incidence.

For the oblique incidence $\theta =15^{\circ }$, we performed measurements of polarization dependence of the remagnetized area. The results are presented in Figure A2 and show that the effect is polarization-insensitive except for the difference arising from the absorption in the GdFeCo film.
